# Intergenerational Educational Pathways and Self-Rated Health in Adolescence and Young Adulthood: Results of the German KiGGS Cohort

**DOI:** 10.3390/ijerph16050684

**Published:** 2019-02-26

**Authors:** Julia Waldhauer, Benjamin Kuntz, Elvira Mauz, Thomas Lampert

**Affiliations:** Robert Koch Institute, Department of Epidemiology and Health Monitoring, Berlin, Germany; b.kuntz@rki.de (B.K.); e.mauz@rki.de (E.M.); t.lampert@rki.de (T.L.)

**Keywords:** intergenerational mobility, health inequalities, education, transitions, self-rated health, young adulthood

## Abstract

Health differences in social mobility are often analysed by income differences or different occupational positions. However, in early adulthood many young people still have very diffuse income situations and are not always fully integrated into the labour market despite many having finished school. This article focusses on the link between intergenerational educational pathways and self-rated health (SRH) among young adults considering their SRH in adolescence. The data source used is the German KiGGS cohort study. The analysis sample comprises 2175 young people at baseline (t0: 2003–2006 age 14–17) and first follow-up (t1: 2009–2012 age 19–24). Combining parent’s and young people’s highest school degree, the data can trace patterns of intergenerational educational pathways (constant high level of education, upward mobility, downward mobility, constant low level of education). Young people’s SRH was recorded at t0 and t1. During adolescence and young adulthood, participants were less likely to report poor SRH if they had a constant high intergenerational education or if they were upwardly mobile. The differences were particularly striking among young adults: average marginal effects (AME) for poor SRH showed much higher risk among downwardly mobile compared to peers with an intergenerational constant high education (AME: 0.175 [0.099; 0.251]), while the upwardly mobile had a significantly lower risk for less than good SRH than peers with an intergenerational constant low level of education (AME: −0.058 [−0.113; −0.004]). In the context of great societal demands and personal developmental needs, educational differences in health tend to increase in young adulthood. Public Health should pay more attention to educational and health inequalities in young adulthood.

## 1. Introduction

Adolescence and young adulthood are significant life phases for personal and health development. The transition from one life phase to the other often opens up opportunities, but it can also entail health risks [1,2]. Young people grow increasingly independent when making health-related decisions and adopting modes of behaviour [3,4,5,6]. Furthermore, social and societal demands increase during the transition to young adulthood. For most young people it is a transition to training and working life [7]. Frequently this involves more autonomy and responsibility, greater demands on personal development, adaption to new life environments [8,9,10], sometimes with consequences for health [11,12,13,14]. 

How well young people cope with these demands will have a long-term impact on their health and is closely linked to their social background [1,11,12,15,16]. Health inequalities have been studied internationally for many years, also among young people [17]. Youths from socially better-off families can often handle developmental tasks better than those from socially disadvantaged families. The lower the family of origin’s socio-economic position, the more often people are already exposed to health risks at a young age and the fewer resources they have at their disposal that might help them to cope with these demands [16,17,18,19]. Based on the assumption that teenage years are formative for health in young adulthood [15,20,21], educational and health trajectories are an important topic for public health.

As in many other countries, educational participation and educational achievements are closely linked to social origin in Germany [22,23,24,25,26]. The allocation into different tracks after elementary school takes place very early. Parents and teachers decide on the educational paths of the young students. Four general types of schools offer educational programs of varying length, depth and emphasis. A distinction is made between a basic and an intermediate type of secondary school. The most advanced grammar school (Gymnasium) leads to the examination, which qualifies for higher education. A fourth type does not fit exactly into this hierarchy. It offers qualification in all three tracks mentioned above. This early tracking usually takes place at the age of 10 to 12 years. It shapes educational opportunities and future perspectives on the labour market. This practice of separation continues in a socially selective training system in Germany [27]. Social selection is present in the German education system far beyond the school age. This practice of social selection has no influence on the formal access of young people to health care in Germany. As a rule, all young people between the ages of 0 and 18 are initially insured by their families as a result of statutory health insurance. They are also insured during their education and study by their families or through training centres or employers. For unemployed young people, the employment agency or family insurance guarantees access to the health system. Nevertheless, there are significant health differences between the social classes.

There is a growing acceptance of the hypothesis that a person’s education is one of the most important health determinants for young people [24,28,29] and for all phases of life [30,31]. Even at an early age, educational differences can be reflected in people’s general and mental health and health-related behaviour [29,32]. In Germany, teenage boys and girls who receive a high-level school education are less likely to smoke [33,34]. They are exposed to fewer psychological risks, have fewer behavioural problems and are more often active in sports [35]. They rate their health and quality of life better frequently [36] and have fewer emotional problems [37] than peers with a low-level school education. 

Educational upward mobility in industrialised countries is more frequent than ever before. It is regarded as a consequence of educational expansion [38,39]. Upward social mobility is often seen as an opportunity in terms of one’s own social position and health [40,41]. For adults, the health-promoting effects of upward social mobility, and higher health risks in the case of downward social mobility, are well documented internationally [40,41,42,43,44,45,46]. Researchers try to explain this association through accumulation [47], the substitution of life circumstances [48,49], unfulfilled expectations [50] or health selection [51,52]. In addition, there are studies focussing on health of young populations regarding educational transmissions between adolescents and their parents. Studies have shown that adolescents who are likely to exceed their parents’ formal level of education live healthier lives. They have a lower risk of smoking, being overweight or obese [53,54], and report better health and life satisfaction than their peers who do not exceed their parents’ education [55,56,57]. 

The link between health and intergenerational education is less studied in young adulthood, especially for educational upward mobility and for young woman. As societal demands grow during the transition from adolescence to young adulthood, the protective effect of educational resources also might increase. However, there is little scientific evidence of this dynamic in Germany. International comparisons show a higher mortality rate among 19 to 29 year-old Belgians with downward social mobility [58] and associations with health related lifestyles for young Australians [43]. Further studies focusing exclusively on downwardly mobile young men also document higher rates of depression and suicidality among 19-year-old Swiss [59], an increased risk of alcoholism among 25 to 34 year-old Swedes [60] and an increased risk of drug use among 25 to 32 year-old Americans whose social position was declining [61]. Up to now, there has been no research on the link between patterns of intergenerational educational pathways and self-rated health (SRH) regarding both young men and women and their SRH in adolescence. The aim of this study was therefore to answer the following research questions:

1. Are there differences in SRH among young adults along their intergenerational educational pathways?
2. Does the association between SRH and intergenerational educational pathways among young adulthood change if SRH is considered during adolescence?
3. Are there differences in SRH and intergenerational educational pathways between young women and men?

## 2. Materials and Methods 

### 2.1. Study Design, Study Population and Weighting

The analyses are based on anonymised data of the first two surveys of the German KiGGS cohort [62]. In the “National Health Interview and Examination Survey for Children and Adolescents” (KiGGS) 0 to 17 year-old participants are followed up into adulthood. KiGGS is part of the national health monitoring conducted by the Robert Koch Institute and funded by the German Federal Ministry of Health. All studies at the Robert Koch Institute are subject to strict compliance with data protection regulations, the EU Basic Data Protection Regulation (DSGVO) and the Federal Data Protection Act (BDSG). The Ethics Commission of the Charité Universitätsmedizin Berlin has supervised the KiGGS Baseline Survey (No. 101/2000) and KiGGS Wave 1 (No. EA2/058/09) approved the studies. Participation in the KiGGS studies was voluntary. The participants or their legal guardians were informed about the objectives and content of the studies and the data protection plan. They gave their written consent.

KiGGS consists of repeated representative cross-sectional surveys and the KiGGS cohort as a longitudinal component [63]. The KiGGS Baseline Survey was conducted as an examination and interview survey from 2003 to 2006 (t0) in a total of 167 randomly selected cities, towns and municipalities in Germany. Children and adolescents between the ages of 0 and 17 were randomly selected from the official population registers of these municipalities stratified according to age cohorts [64]. The overall response rate was 66.6%. The total sample comprised 17,641 participants (8985 boys and 8656 girls). A weighting factor was calculated which corrects the deviations of the net sample from the population structure (as per 31 December 2004) with regard to age, gender, region, nationality and parents’ educational level [64]. 

All the participants in the KiGGS Baseline Survey were invited to participate in the first follow-up survey KiGGS Wave 1 (2009–2012, t1). A total of 11,992 of the former participants (5914 males, 6078 females aged 6 to 24 in the meantime) agreed to take part again. KiGGS Wave 1 was conducted as a telephone-based interview. A weighting factor based on sociodemographic characteristics [62] was calculated to compensate for selective willingness to participate again. 

Only young people who were 19 to 24 years old during KiGGS Wave 1 (t1) and correspondingly 14 to 17 years old during KiGGS Baseline (t0) were included in the analyses. In addition, participants were limited to those who attended a regular German school and had provided information on their own education and the education of their parents. From the total sample of 2175 participants (1010 males, 1165 females) 15.72 % were excluded because of missing information on educational background, SRH and sociodemographic factors.

### 2.2. Variables and Measurement

#### 2.2.1. Outcome Variable: Self-Rated Health 

SRH is a global measure of health, reflecting diseases and everyday complaints. It covers both the personal and social dimensions of physical and psychosocial health and well-being. Young people with poor SRH have a higher risk of chronic physical diseases and mental impairments during the further life course [21,65]. Both as adolescents (t0) and in young adulthood (t1), the cohort participants answered the question: “How do you assess your health in general?”, using a 5-step scale ranging from “very good”, “good” and “fair” to “poor” and “very poor”. For simplicity these groups were dichotomised into good (the first two categories) and fair/poor (the other three categories).

#### 2.2.2. Exposure: Own Education, Parents’ Education and Intergenerational Educational Pathways

Information on young people’s education was collected and dichotomised on the basis of the highest school-leaving certificate attained at t1. The young people were subsequently classified according to their level of school education as having achieved a ‘high-level’ education (with a subject-specific or general university entrance qualification) or a ‘low-level’ education (all school-leaving qualifications below this level, including no school-leaving certificate at all).

The dichotomisation of the education of the parents (high-level vs. low-level) was also based on information provided on their highest school-leaving certificate. The highest educational level of both parents or the highest educational level of the one parent with whom the young people were living at the time was recorded at t0. A high level of parents’ education was assumed if at least one parent (mother or father or both) had a subject-specific or general university entrance qualification. Parents, who acquired a high-level certificate at an advanced secondary school (EOS) in the former GDR, or an equivalent school-leaving certificate in a country other than Germany, were also assigned to the group with a high level of education. All other parents were assigned to the group with a low level of education.

In order to study intergenerational educational pathways, the dichotomised educational variables of young adults were compared with those of their parents [54,57]. In this way, we distinguished four patterns of intergenerational educational pathways: constant high education (parents’ education high, own education high), upward mobility (parents’ education low, own education high), downward mobility (parents’ education high, own education low), and constant low education (parents’ education low, own education low). 

#### 2.2.3. Other Model Variables: Sociodemographic Factors

Additional sociodemographic information collected in KiGGS Baseline Survey (t0) was included in the analyses as control variables. The trait “migration background” was ascribed to those participants who migrated to Germany herself/himself, or in case that at least one of their parents was not born in Germany, had migrated to Germany, or had a nationality other than German [66]. The region of residence was determined based on the cities, towns and municipalities where the sampling took place. The household’s equivalised income was calculated based on the parents’ monthly net income and the number of people living permanently in the household [67]. The household equivalised income was logarithmised for the analyses. Furthermore, the young peoples’ age, sex and the age of their parents were included in the analyses. An interaction of intergenerational educational mobility and sex was included in the regression analysis to account for possible gender bias in intergenerational educational mobility.

### 2.3. Statistical Analyses

We used descriptive statistics to analyse educational transmissions between young people and their parents. Educational transmissions were compared with SRH in adolescence (t0, ages 14 to 17) and SRH in young adulthood (t1, ages 19 to 24). Using binary logistic regression models, we estimated average marginal effects (AME). In the first model (M1) we analysed SRH of young adults and the intergenerational educational pathways including the control variables. Additionally, in the second model (M2) we controlled for SRH at the age of 14 to 17. 

We performed all analyses for the total, male and female population. Proportions and AME were reported with 95% confidence intervals (95% CI). For the statistical analyses we used Stata/SE 15.1. To compensate for differences between the cohort participants and those who did not take part again we used a calculated longitudinal weighting factor for the entire analysis [62]. In order to take the weighting factors into account, all the analyses were carried out with the survey procedures (svy).

## 3. Results

### 3.1. Sample Description

Table 1 presents the analysis sample and the distribution of the variables considered separately for female and male (Table 1).

### 3.2. Descriptive Analysis: Patterns of Intergenerational Educational Pathways and Self-Rated Health

Overall, about 71.3 % of parents had no high-level school-leaving certificate and 28.7 % had a high-level school education. About half of the young people (50.3 %) reached a high educational level, the other half a low level of education (49.7%). A high parental education level was more likely to be continued in a high level of school education in the next generation (80. 3 %). 19.7 % did not reach their parents’ high educational level and were therefore regarded as downwardly mobile. By contrast, 60.3 % of young people whose parents had a low level of education remained at this educational level while 39.7 % exceeded their parent’s educational level and were thus upwardly mobile. 

**Figure 1:**
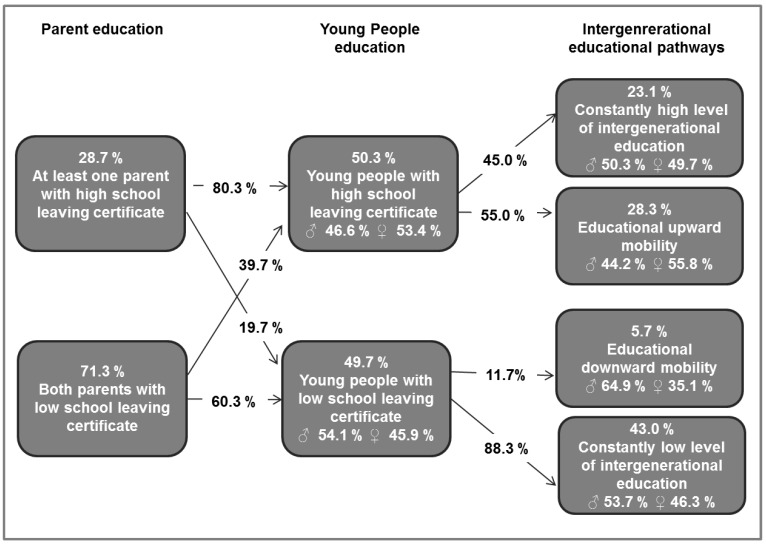
Intergenerational educational pathways: comparing the highest school-leaving certificates of young people and their parents.

In total, 23.1% of the sample population had a constant high educational level (parents’ education high, own education high), 5.7 % were downwardly mobile (parents’ education low, own education high), 28.3 % were upwardly mobile (parents’ education low, own education high), and 43.0 % showed a constant low level of education (parents’ education low, own education low). Young women were slightly more frequently upwardly mobile. Young men were more frequently to be found in the group with a constant low level of education and among the downwardly mobile (Figure 1).

The general analysis of SRH by age and gender (results are not shown here) indicated that 15.2% of the cohort participants in adolescence (t0, aged 14 to 17) assess their SRH as fair/poor. In young adulthood (t1, aged 19 to 24), the percentage is 13.6%. Adolescent girls (t0) stated fair/poor SRH slightly more frequently than boys (15.9% vs. 14.5%). This tendency continued among young women compared to young men (t1: 14.3% vs. 12.9%).

**Figure 2:**
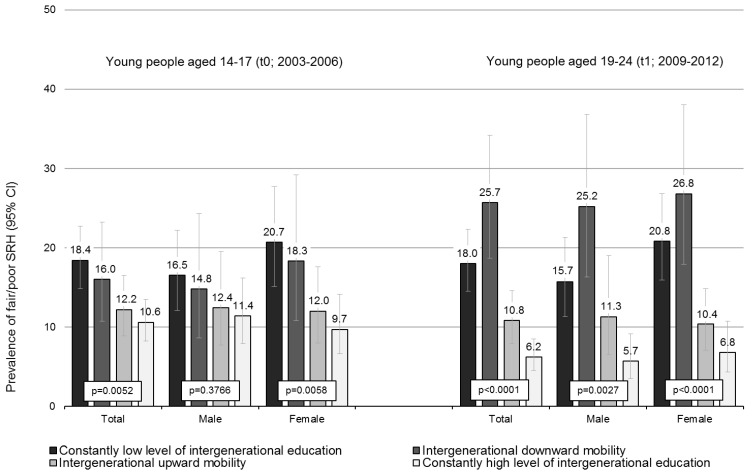
Proportions of fair/poor SRH during adolescence (t0) and young adulthood (t1) regarding intergenerational educational pathways (t1); *p*-values by Pearson’s Chi-square test.

The descriptive analysis of SRH and patterns of intergenerational educational pathways revealed differences in SRH to the disadvantage of downwardly mobile young adults and those with an intergenerational constant low level of education. One in four downwardly mobile young adults reported fair/poor SRH, while regarding young adults with an intergenerational constant low level of education, it was almost one in five. The differences were similarly pronounced and significant in both young men and women (Figure 2, right-hand side).

To assess whether these differences already begin to become apparent at a younger age, the patterns of intergenerational educational pathways were also compared with the information on SRH in adolescence (t0, 14 to 17). Disadvantages in SRH already occurred during adolescence, especially for those who were going to have an intergenerational low level of education (Figure 2, left-hand side). 

### 3.3. Multivariate Analysis: SRH and Patterns of Intergenerational Educational Pathways in Young Adulthood

The multivariate results confirmed a strong correlation between SRH and intergenerational educational pathways to the disadvantage of the downwardly mobile group and young adults with an intergenerational constant low level of education. The comparison between Model 1 and 2 revealed that those differences can be partly explained through SRH during adolescence (Table 2). AME decreased for almost every pattern of intergenerational educational pathways when adjusting for SRH in adolescence (M2). However, this was the case only to a very small extent.

The comparison of the AME for different patterns of intergenerational educational pathways with both reference groups (constantly low and constantly high) showed strong differences between the groups. After the adjustment for sociodemographic aspects and SRH in adolescence (M2), the risk of fair/poor SRH reduced for the group with constantly high education (AME −0.108 [−0.157; −0.060]) and upward mobility (AME −0.058 [−0.113; −0.004]) if the reference group was constantly low. The group with downward mobility did not differ on a significant level. However, the downward mobile young adults tended to have a higher risk of a fair/poor SRH (AME 0.067 [−0.018; 0.152]) than the group with constantly low education. 

Compared to the reference group with constantly high education all other groups of intergenerational educational pathways revealed significantly higher risks of poor/fair SRH. The downwardly mobile 19 to 24 year-olds showed the highest risk of fair/poor SRH (AME 0.175 [0.099; 0.251]), followed by the constantly low (AME 0.108 [0.060; 0.157]) and the upward mobile young adults (AME 0.050 [0.010; 0.091]). 

With two exceptions (downward mobility and upward mobility compared to reference group constantly low) young men showed slightly higher differences between the educational groups than young women. However, the tendencies of AME showed the same patterns for both male and female in all comparisons. Only between the upward mobile young men and women there were small differences regarding the significance of AME compared to both reference groups.

## 4. Discussion

The results showed a strong transmission of educational levels within families. Young adults most often achieved the same school leaving certificate when their parents had a high level of school education. Nevertheless, around one third of the analysed sample was upwardly (28.3 %) and downwardly (5.7 %) mobile. Constantly low educated and downwardly mobile young women and men stated fair/poor SRH much more frequently than the other two groups. Even if upwardly mobile young men and women rated their health more often as good or very good compared to those with constantly low education, they did not open up to the group with constantly high education. Downwardly mobile young adults stated fair/poor SRH more often than those with constantly low education. But that difference was not on a significant level. SRH in adolescence explained our findings only to a very small extent. Young women and men showed similar effects of intergenerational educational pathways on SRH. For young men, the findings were slightly more pronounced.

In line with our findings, other studies reported a large extent to which the education of the following generation is influenced by parents’ education [68,69]. Further studies proved that this is not a German [69,70,71], but an international phenomenon [24]. The educational expansion may be one reason for the large difference between young adults and the education of their parents. Higher education becomes more common in modern countries. The orientation and course of a person’s life has never been tied to educational processes so intensively and enduringly as in today’s societies [72,73]. However, if a high level of education is increasingly required, it can also mean a risk for people who do not meet this requirement. Especially for children from disadvantaged families, upward mobility in educational attainment is still rare [74].

With regard to health differences and intergenerational educational mobility, our findings complement and extend the current state of research. In line with our results, some studies pointed to health differences depending on intergenerational educational pathways in Germany, but only for 12 to 17 year old adolescents [28,53,54,55,57]. As an extension of this research, we based our study on school-leaving certificates actually attained and thus trace social mobility with regard to formal completed school education. Internationally, few studies have taken into account the life phase of young adulthood focused on here, and only reported results on young men in most cases. These studies related not only to educational mobility but mainly to intergenerational differences in labour market positions. They referred higher risks for depression and suicidality, alcohol and drug consumption among upwardly mobile adults [59,60,61]. Another study reported the subjective assessment of potential educational mobility—in this case an anticipated downward social mobility among young males—as to be associated with adverse health-related modes of behaviour [75].

Although comparable studies are rare, there are different theoretical frames to which our results can be related. The accumulation theory—in this case accumulation of educational resources—says that advantages in initial resources can predict several further advantages in life [47]. Thus, success on the labour market and other opportunities for life are closely linked to the education of people in modern societies. Our findings support that, overall, young people with a higher level of education report fair/poor SRH much less frequently than those with a low educational level. This might result out of better economic prospects in the group with higher education.

According to the substitution of life circumstances [49], some researchers state that young people’s social target position is more important for health than their social starting position [43,54,56]. In this theoretical frame it is said that people frequently adjust to the prevalent modes of behaviour when they change to a new social position. People who move down from a higher position frequently suffer from probably less favourable life opportunities. This can be further aggravated by a component of negative attribution and disappointed expectations—one’s own and those of family and society [48,50]. By contrast, social upward mobility can be seen as a chance for healthier lives. Our findings indicated that upwardly mobile young adults stated better SRH compared to the group with constantly low education. But they did not open up to the group with constant high education. This is in line with other studies revealing that upwardly mobile people did not reach the health status of the group they joined. Further studies showed that social mobility can even have bad implications for health [76,77]. Thus, the theoretical frame for the substitution of life circumstances seems limited. One reason might be that young adults who have completed school often still rely on their parents’ financial resources. Those resources are highly correlated with the educational attainment of their parents. Since young adults face longer periods of education, they can depend even longer on their parents’ resources for example to pay tuition fees or living expenses during vocational and university training. Other explanations are due to aspects of area deprivation or status-based identity [76]. Studies showed a strong correlation of the subjective perception of one’s own social position and health [78,79]. In this context, it would be important to examine how young people who are upwardly or downwardly mobile assess their subjective social status compared to those who have remained in the group of origin. 

Based on the theory of reverse causation or social selection by health differences, other studies revealed that young people with health problems had greater difficulties in coping with the demands of school and with those of developing into young adulthood. Absenteeism due to illness and reduced performance due to mental or health problems can seriously disrupt educational pathways [80,81]. In our study, we found a very small reduction of the association of SRH and intergenerational educational mobility in young adulthood after adjustment for SRH in adolescence. It is very likely that there are some stronger but unrecognized additional factors that explain some of the observed associations. Cognitive and non-cognitive skills may be relevant to this context [82] or the economic situation of young adults themselves.

Gender-related differences were small in our study. They cannot be fully allocated to other studies. We found two other studies that referred to risk behaviour and mortality among both sexes and considered about the same age that we focused on [43,58]. Although there were differences in significance, our study showed that both young women and men stated better SRH when they were upwardly mobile compared to the group with constantly low education. Both upwardly mobile women and men did not achieve the same rate of good SRH compared to the group with a constantly high level of education. This is not in line with the findings of Gall, Abbott-Chapman, Patton, Dwyer and Venn [43], who found higher chances for healthy lifestyles for upwardly mobile young women than for men. Their results, as well as our own findings, contrast with those of De Grande, Vandenheede and Deboosere [58], who found the highest level of mortality among downward mobile young women. While our results suggest that young men show the most marked differences in SRH and intergenerational educational mobility, these studies differ strongly in terms of the study sample and the outcomes.

As in all cohort studies, a systematic distortion due to loss-to-follow-up in the KiGGS cohort cannot be completely ruled out. The KiGGS study applied various strategies [83] to improve the response rates of hard-to-reach groups. Nevertheless, a form of selection bias is conceivable. This is particularly to be expected in the lower education groups. All analyses were therefore calculated with the longitudinal weighting factor created for the KiGGS cohort. The follow-up was adjusted to the baseline study composition on the basis of sociodemographic aspects [62].

The analyses were based on self-rated information. In the KiGGS Basline Survey, information was collected by means of written questionnaires and in KiGGS Wave 1 by telephone interviews. Sociodemographic information proves to be relatively stable between different survey methods [84]. Differences in response behaviour on SRH at two time points using different survey methods cannot be ruled out [85]. This must be taken into account when comparing SRH in adolescence and young adulthood.

The education indicator used here, which was based exclusively on a completed school education, is possibly limited. At the age of 19 to 24, however, most of the cohort participants had completed their secondary education. School-leaving certificates were therefore the only comparable indicator among young adults and their parents. 

Furthermore the analysis did not include other explanatory variables that could explain the association of SRH and intergenerational educational mobility. Next to cognitive skills and personal resources the financial background of young adults may be informative in this context.

With regard to the sample population, it should be noted that the downwardly mobile group is relatively small compared to the other patterns of intergenerational educational pathways.

## 5. Conclusions

The transition from adolescence to young adulthood is a significant phase for young peoples’ health. It is a time of many personal changes and important decisions. Since the period of emerging adulthood is increasingly prolonged and often characterised by instability and uncertainty [8,86], there are already calls from psychotherapeutic and psychiatric care for the services offered to focus more on this phase of life [8]. Moreover, it becomes increasingly difficult to reach young people during young adulthood. Young peoples’ life pathways develop in very different directions during this period and this should be considered when developing support structures. In our view, also public health scientist and professionals in educational and training institutions should deal in more detail with this life phase.

For this age group our study showed large differences in SRH along intergenerational educational pathways. Young adults often do not yet fully participate in the labour market. Their financial situation is sometimes difficult to compare. That’s why educational differences are an appropriate way to take into account social differences in this phase of life. Investigations in educational differences in health should not only include the educational background of the study population. Especially in connection with the education of their parents, the intergenerational educational level of people seems to be important for the production and reproduction of health inequalities. 

Due to various methods for measuring intergenerational mobility in the international research landscape, further research is needed. The proposal made here is to compare education levels, as young people are often not yet fully integrated into the labour market. For example, conflicting observations among young adults about the relationship between health and upward and downward mobility should be elucidated. The ambiguous gender differences should also be further investigated. In order to better understand the correlation of SRH and intergenerational educational mobility, it would be helpful to analyse other explanatory factors such as cognitive and personal resources, the subjective social status as well as the financial situation of young adults in subsequent analyses. 

## Figures and Tables

**Table 1:** Description of the study sample (n = 2175).

Characteristics	Male % (n = 1010)	Female % (n = 1165)
**SRH t0**		
Good	85.51 (1632)	84.08 (1515)
Fair/poor	14.49 (254)	15.92 (302)
**SRH t1**		
Good	87.09 (895)	85.66 (1010)
Fai/poor	12.91 (115)	14.34 (155)
**Age: Adolescence t0**		
Mean	15.49 (15.39)	15.43 (15.48)
SD	0.04 (0.03)	0.04 (0.03)
**Age: Emerging adulthood t1**		
Mean	21.57 (21.44)	21.52 (21.56)
SD	0.05 (0.04)	0.04 (0.03)
**Parental education t0**		
High	30.15 (647)	27.20 (597)
Low	69.85 (1074)	72.80 (1056)
**Young people’s education t1**		
High	46.56 (562)	54.00 (739)
Low	53.44 (420)	46.00 (400)
**Intergenerational educational pathways t0 and t1**		
Constant high school education	22.84 (318)	23.38 (353)
Educational upward mobility	24.57 (223)	32.10 (350)
Educational downward mobility	7.24 (89)	4.05 (65)
Constant low school education	45.35 (301)	40.47 (300)
**Age: Mother t0**		
Mean	43.20 (42.67)	42.49 (42.41)
SD	0.23 (0.12)	0.21 (0.12)
**Age: Father t0**		
Mean	46.58 (45.70)	45.72 (45.38)
SD	0.31 (0.15)	0.29 (0.16)
**Migration Background t0**		
Yes	23.66 (415)	19.09 (350)
No	76.34 (1488)	80.92 (1479)
**Region of residence t0**		
Eastern (newly formed German states incl. Berlin)	20.33 (623)	14.17 (643)
Western (old western German states)	79.67 (1281)	85.83 (1.189)
**Equivalence income t0**		
Mean	1123.06 (1184.22)	1109.75 (1163.71)
SD	18.98 (13.44)	22.03 (13.28)

% weighted according to data on the residential population of Germany, 31 December 2004 & 2010; (n) unweighted; SD = standard deviation; t0 KiGGS Baseline Survey (2003–2006); t1 KiGGS Wave 1 (2009–2012).

**Table 2:** AME based on logistic regression analysis of the relationship between fair/poor SRH of young adults and intergenerational educational pathways (Ref. constantly high education and constantly low education).

Intergenerational Educational Pathways	M1			M2		
	Total	Male	Female	Total	Male	Female
Constantly high	−0.114 *** [−0.164; −0.065]	−0.119 ** [−0.189; −0.050]	−0.110 ** [−0.179; −0.040]	−0.108 *** [−0.157; −0.060]	−0.119 ** [−0.187; −0.051]	−0.099 ** [−0.168; −0.030]
Downward mobility	0.073 [−0.017; 0.163]	0.068 [−0.051; 0.186]	0.079 [−0.042; 0.200]	0.067 [−0.018; 0.152]	0.066 [−0.046; 0.178]	0.067 [−0.045; 0.180]
Upward mobility	−0.065 * [−0.120; −0.010]	−0.053 [−0.141; 0.035]	−0.078 * [−0.144; −0.012]	−0.058 * [−0.113; −0.004]	−0.047 [−137; 0.042]	−0.071 * [−0.135; −0.008]
Constantly low (Ref.)	Ref.	Ref.	Ref.	Ref.	Ref.	Ref.
Constantly high (Ref.)	Ref.	Ref.	Ref.	Ref.	Ref.	Ref.
Downward mobility	0.187 *** [0.105; 0.269]	0.187 ** [0.072; 0.302]	0.189 ** [0.078; 0.299]	0.175 *** [0.099; 0.251]	0.185 ** [0.076; 0.294]	0.166 ** [0.066; 0.266]
Upward mobility	0.049 * [0.010; 0.088]	0.067 * [0.001; 0.132]	0.031 [−0.019; 0.082]	0.050 * [0.010; 0.091]	0.072 * [0.003; 0.140]	0.027 [−0.023; 0.078]
Constantly low	0.114 *** [0.065; 0.134]	0.119 ** [0.050; 0.189]	0.110 ** [0.040; 0.179]	0.108 *** [0.060; 0.157]	0.119 ** [0.051; 0.187]	0.099 ** [0.030; 0.168]

[95% CI] * (*p* < 0.05), ** (*p* < 0.01), *** (*p* < 0.001). M1: Fair/poor SRH (t1) and intergenerational educational pathways, adjusted for young people’s and parents’ age, migration background, region of residence and logarithmic household equivalised income and interaction of intergenerational educational mobility and sex. M2: Fair/poor SRH (t1) and intergenerational educational pathways, adjusted for young people’s and parents’ age, migration background, region of residence and logarithmic household equivalised income and interaction of intergenerational educational mobility and sex, controlling for SRH during adolescence (t0).

## Abbreviations

AME	Average marginal effects
CI	Confidence interval
SRH	Self-rated health
